# Comparison of different statistical models for the analysis of fracture events: findings from the Prevention of Falls Injury Trial (PreFIT)

**DOI:** 10.1186/s12874-023-02040-1

**Published:** 2023-10-02

**Authors:** Anower Hossain, Ranjit Lall, Chen Ji, Julie Bruce, Martin Underwood, Sarah E. Lamb

**Affiliations:** 1 Warwick Clinical Trials Unit, University of Warwick, Coventry, UK; 2 Institute of Statistical Research and Training (ISRT), University of Dhaka, Dhaka, Bangladesh; 3 University Hospital Coventry and Warwickshire NHS Trust, Clifford Bridge Road, Coventry, UK; 4 University of Exeter, St Luke’s Campus, Exeter, UK

**Keywords:** Fracture counts, Poisson regression, Negative binomial regression, Count data, Excess zero counts

## Abstract

**Background:**

Fractures are rare events and can occur because of a fall. Fracture counts are distinct from other count data in that these data are positively skewed, inflated by excess zero counts, and events can recur over time. Analytical methods used to assess fracture data and account for these characteristics are limited in the literature.

**Methods:**

Commonly used models for count data include Poisson regression, negative binomial regression, hurdle regression, and zero-inflated regression models. In this paper, we compare four alternative statistical models to fit fracture counts using data from a large UK based clinical trial evaluating the clinical and cost-effectiveness of alternative falls prevention interventions in older people (Prevention of Falls Injury Trial; PreFIT).

**Results:**

The values of Akaike information criterion and Bayesian information criterion, the goodness-of-fit statistics, were the lowest for negative binomial model. The likelihood ratio test of no dispersion in the data showed strong evidence of dispersion (chi-square = 225.68, *p*-value < 0.001). This indicates that the negative binomial model fits the data better compared to the Poisson regression model. We also compared the standard negative binomial regression and mixed effects negative binomial models. The LR test showed no gain in fitting the data using mixed effects negative binomial model (chi-square = 1.67, *p*-value = 0.098) compared to standard negative binomial model.

**Conclusions:**

The negative binomial regression model was the most appropriate and optimal fit model for fracture count analyses.

**Trial registration:**

The PreFIT trial was registered as ISRCTN71002650.

## Introduction

Reducing fractures in older people is a public health priority. Few community-based clinical trials of fracture prevention have been sufficiently powered to show reductions in fractures [1, 2]. Inevitably, sampling participants from the general population means that only a very small proportion of the target population sustain a fracture over the period of observation. For a large UK cluster randomised controlled trial (RCT) (63 general practices, *n* = 9803 participants) investigating a screen and treat approach offering falls prevention interventions to prevent falls and fractures, we required an appropriate statistical strategy for the trial analyses. The Prevention of Falls Injury Trial (PreFIT) methods and results have been reported in detail elsewhere [2–4].

Statistical methods specifically for analysing fracture counts are less common in the literature, compared to methods for the analyses of falls. Fracture data are distinct from other count data for a number of reasons, including: (a) the majority of data are positively skewed with a large proportion of zero events; (b) multiple fractures can occur at one anatomical site at the same time (e.g. hand, foot or rib fractures); (c) fracture events can be recurrent, in that some people can fracture repeatedly over a period of time, and (d) some have multiple injurious falls with multiple fracture events. A Cochrane systematic review of interventions to prevent falls in older people identified 62 trials that had used inappropriate statistical analysis methods to test intervention efficacy [5]. Some trials incorrectly assumed normality for distribution of falls data and used linear regression and t-tests, whereas other trials used analysis of variance. The number of falls or fractures over time are count data, often positively skewed with a large proportion of zeros. There are several recommended methods for analysing count data, namely, Poisson regression, generalised Poisson regression, negative binomial regression, hurdle regression, and zero-inflated regression [6]. However, it is not clear which statistical method works best when count data are positively skewed with a large proportion of zero counts.

Robertson et. al. [7] compared the performance of negative binomial regression with two different survival analysis methods (Andersen-Gill and marginal Cox proportional hazards regressions) using data from two randomised trials testing the Otago home exercise programme to prevent falls. The authors recommended using negative binomial regression to measure the efficacy of fall prevention trials. However, the research team did not compare the performance of negative binomial regression to other methods of analysing count data. The superseded Cochrane systematic review [8] identified that many trials used negative binomial regression to compare the intervention between treatment arms, as recommended by Robertson [7]. Guthrie et. [9] investigated the performance of different statistical methods for skewed counts using genetic data and showed that negative binomial regression performed well compared to the other methods for count data. However, it was unclear how well the negative binomial distribution would perform when data have more than 95% zero counts. In this study, we undertook a secondary analysis to examine the utility of different statistical models that take account of count data, using the example of fracture events. We propose these issues should be considered in future analyses of fracture outcomes in clinical trials.

The paper is organised as follows. We firstly present a brief introduction to Poisson distribution, Poisson regression, negative binomial regression, hurdle and zero-inflated regression models. We then present our analyses using the PreFIT dataset, describing the goodness-of-fit statistics and results for each statistical model. Finally, we discuss the implications of our findings.

## Data

We used anonymised data from a completed, large UK-based, three-arm pragmatic cluster RCT, PreFIT [10]. The aim of the trial was to compare the clinical and cost-effectiveness of three alternative fall prevention interventions: advice only, advice with exercise, and advice with multifactorial falls prevention (MFFP), in adults aged 70 years and older. Sixty-three general practices (total 9803 participants) were randomised into three intervention arms: 21 practices [3223/9803 (33%) participants] to advice only; 21 practices [3279/9803 (33%)] to exercise, and 21 practices [3301/9803 (34%)] to MFFP. The primary outcome was fractures over 18 months, expressed as the fracture rate per person per 100 years [2]. We ascertained fractures using multiple methods, from a) UK National Health Service (NHS) Digital Hospital Episode Statistics (HES) (Admitted Patient Care and Accident and Emergency data); b) general practices using consultation codes, x-ray reports and hospital correspondence; and c) participant self-report collected by postal survey. All fracture events were independently confirmed by a panel of clinicians blinded to treatment allocation.

## Overview of analytical approaches

### The poisson distribution

The Poisson distribution is commonly used to model the number of events (count data) over a specified period [11]. The Poisson distribution assumes that (i) the probability of an event occurring in a distinct and non-overlapping interval of time is independent of the probability of an event occurring in other intervals, and (ii) the probability of an event occurring in an interval is proportional to the length of the interval [12]. Both the mean and the variance of Poisson distribution are equal to the only parameter of the distribution, which is calculated as the rate of the occurrence of the events.

### Poisson, negative binomial, hurdle, and zero-inflated regression models

The Poisson regression, negative binomial regression, hurdle regression, and zero-inflated regression models are generalised linear models widely used to model count data. We briefly discuss the assumption of each of these models (Table 1).

**Table 1:** Possible statistical models and assumptions for count data

Model	Assumptions
Poisson regression	Mean and variance are equal
Negative binomial regression	Overdispersion in the data, where variance is greater than mean
Zero-inflated model	Two sources of zeros: “structural” and “sampling”. Structural zeros are due to some specific structure in the data and sampling zeros are due to Poisson or negative binomial distribution
Hurdle model	All zeros are from a “structural” source and all positive values are from a “sampling source”

The Poisson regression model assumes that the mean and variance of the outcome are the same [12], which is a very strong assumption and is unlikely to hold in practice. With count data, it is commonly observed that the variance of the outcome is greater than the mean of the outcome, known as over-dispersion. Negative binomial regression is widely used for over-dispersed count data [13, 14]. Sometimes count data contain excess zero counts relative to data from a standard Poisson distribution. For example, the number of GP (general practice) visits for seasonal flu within a population could be inflated by zero counts by those who did not have flu. In such cases, there are two types of zero counts in the data: (i) the number of GP visits is zero (certain zeros) for those who did not have flu and thus did not visit their GP, and (ii) the number of GP visits is zero (sampling zeros) for those who had flu but did not attend their GP. The zero-inflated models are used in such cases. The certain zeros, also known as structural or non-risk zeros, are due to some specific structure in the data as they will always be zero [14, 15]. A logit model is used to predict the probability of these zero counts. Sampling or at-risk zeros, with other positive counts, can be modelled using Poisson or negative binomial regression. The hurdle model assumes that all zeros are structural, that is, certain zero due to some structure of the data, and the positive values are due to sampling [16, 17].

### Presence of repeated observations

In a cluster RCT, it is assumed that the observations are more likely to be similar within the same cluster compared to observations in the other clusters [18]. Ignoring such dependencies between the observations in the same cluster will underestimate the standard error of the parameter estimates and, consequently, will result in an inflated type I error rate [19]. One way to account for such intra-cluster dependencies is to include a random effect term into the model, called a mixed effects model. In cohort studies and clinical trials, it is common for participants to have variable follow-up times. In such cases, the natural logarithm of the follow-up periods is included in the model as an offset term to allow us to model counts in proportion to the variable follow-up periods. In clinical trials, individuals or clusters of individuals are randomly allocated to intervention groups. This randomisation ensures that interventions groups are balanced, on average, in terms of baseline covariates. However, this assumption might not be valid with small number of clusters in each group [20]. To account for possible imbalance between the arms, the analysis model is usually adjusted for baseline covariates, which increases the credibility of the trial findings and improves the statistical power [21].

## Data Analysis

### The Prevention of Falls Injury Trial (PreFIT) dataset

In this post-hoc analysis, we applied the four regression models described in Sect. 3.2 to the PreFIT dataset. As described, the primary outcome was the rate of fractures over the 18 months period per person per time unit. The follow-up periods varied by participant. Table 2 shows the distribution of the number of fractures over 18 months by the three intervention arms. Of the 9803 participants, 9424 (96%) participants had no fractures and 379 (4%) participants had at least one fracture over the 18 month follow-up period. The distribution of the number of fractures across the intervention arms were similar. The mean and variance of the number of fractures were 0.05 and 0.07, respectively, which suggests over dispersion in the data.

**Table 2:** Distribution of number of fracture events amongst 9803 participants over 18 months by PreFIT treatment arm

No. of Fractures	Advice N (%)	Exercise N (%)	MFFP N (%)	Total N (%)
0	3113 (96.6)	3153 (96.2)	3158 (95.7)	9424 (96.1)
1	93 (2.9)	104 (3.2)	121 (3.7)	318 (3.2)
2	13 (0.4)	18 (0.6)	17 (0.5)	48 (0.5)
3	2 (0.1)	4 (0.1)	4 (0.1)	10 (0.1)
4	2 (0.1)	0(0)	0(0)	2 (0.02)
6	0 (0)	0(0)	1 (0.03)	1 (0.01)
Total	3223	3279	3301	9803

Figures in parentheses are column percentages, and the sum of column percentages might not be exactly 100 because of rounding

People can experience multiple fractures from one fall. Instead of recording the number of fractures associated with each fall, the trial recorded the number of fall-related fracture episodes over the entire follow up period, which represents the total number of times a participant had fractures due to falling. Table 3 shows the distribution of the number of fracture episodes by three intervention arms. We observed that 9424/9803 (96%) participants had zero fracture episodes, 356/9803 (3.6%) participants had a single fracture episode only, and 23/9803 (0.3%) participants had repeated fracture episodes, that is, more than one fracture episode over 18 months follow up. The number of fracture episodes was evenly distributed across the intervention arms. It was not possible to fit a statistical model to compare fracture episodes due to the small number of participants with repeated episodes (*n* = 23; 0.3%).

**Table 3:** Distribution of fracture episodes in participants by intervention arm

No. of fracture episodes	Advice N (%)	Exercise N (%)	MFFP N (%)	Total N (%)
0	3113 (96.6)	3153 (96.2)	3158 (95.7)	9424 (96.1)
1	102 (3.2)	121 (3.7)	133 (4.0)	356 (3.6)
> = 2	8 (0.3)	5 (0.2)	10 (0.3)	23 (0.3)
Total	3223	3279	3301	9803

Figures in parentheses are column percentages, and  the sum of column percentages might not be exactly 100 because of rounding

### Statistical analysis

As discussed in section Poisson, negative binomial, hurdle, and zero-inflated regression models, the zero-inflated model assumes two sources of zeros (structural and sampling) and the hurdle model assumes certain zeros in the data. In the PreFIT dataset, the zero fracture counts were sampling zeros and we, therefore, did not consider zero-inflated and hurdle models although had 96% zero counts in the data.

A series of four models were fitted: Poisson regression, negative binomial regression, mixed effects Poisson regression, and mixed effects negative binomial regression. We included an offset in each of the models to account for variable follow up periods. Models were adjusted for the baseline covariates of age, gender, GP deprivation score, and natural logarithm of baseline falls count. We adjusted for the natural logarithm of baseline falls count because the distribution of baseline falls count was highly skewed and this approach fitted the model better compared to adjusting for baseline falls count. We added 0.5 to the zero baseline falls count for mathematical convenience. To account for the possible dependency among the observations from the same general practice, we included a random effect for general practices in the mixed effects models. The rate ratios (RaRs) with 95% confidence interval (CI) were calculated to compare the effects of the active intervention arms (exercise or MFFP) versus the control arm (advice). The goodness-of-fit of the models was assessed using Akaike information criterion (AIC) and Bayesian information criterion (BIC). Lower values of AIC and BIC indicate a better fit. We also used the likelihood ratio (LR) test to test the following hypotheses: (i) there was no dispersion in the data, that is, there was no gain of using standard negative binomial regression over standard Poisson regression, (ii) the mixed effects Poisson model fitted the data better compared to the standard Poisson model, and (iii) the mixed effects negative binomial model fitted the data better compared to the standard negative binomial model. We used STATA (version 17) to analyse data.

Table 4 presents the goodness of fit statistics, AIC and BIC, for each of the four considered models. The results show that both AIC and BIC values are the lowest for the negative binomial model, which means the negative binomial model fitted the fracture data better compared with the other three models. The LR test of no dispersion in the data showed strong evidence of dispersion (chi-square = 225.68, *p*-value < 0.001). This indicates that the negative binomial model fitted the data better compared to the Poisson regression model. When we compared the mixed effects Poisson regression model and the standard Poisson regression, the LR test showed strong evidence (chi-square = 11.62, *p*-value < 0.001) that the mixed-effect Poisson model fitted the data better compared to the standard Poisson model.

**Table 4:** Goodness of fit statistic and likelihood test results for model comparisons

Regression Models	AIC	BIC	LR test^e^
Poisson			
Unadjusted	3875.85	3897.42	- -
Adjusted^1^	3528.97	3579.11	
Mixed effects Poisson			
Unadjusted	3866.23	3895.00	Chi-square value = 11.62^b^
Adjusted^a^	3527.10	3584.40	*p*-value < 0.001
Negative Binomial (NB)			
Unadjusted	3652.17	3680.93	Chi-square value = 225.68^c^
Adjusted^a^	3363.62	3420.93	*p*-value < 0.001
Mixed effects NB			
Unadjusted	3652.50	3688.45	Chi-square value = 1.67^d^
Adjusted^a^	3363.62	3420.93	*p*-value = 0.10

*AIC* Akaike Information Criterion, *LR* Likelihood ratio. ^a^Adjusted for baseline covariates age, gender, deprivation score and log of baseline falls count, ^b^LR test of mixed effects Poisson regression vs standard Poisson regression, ^c^LR test of dispersion, ^d^LR test of mixed effects NB regression vs standard negative binomial regression, and ^e^the LR tests results are for the unadjusted models

Finally, we compared the standard negative binomial regression and mixed effects negative binomial models. The LR test showed no gain in fitting the data using mixed effects negative binomial model (chi-square = 1.67, *p*-value = 0.098) compared to standard negative binomial model. In addition, the same adjusted AIC and BIC for the standard negative binomial regression model and mixed-effect negative binomial regression model indicated that there was no clustered structured in the data although the data came from a large, multicentre trial. It is important here to note that one should fit mixed-effect negative binomial regression models first over the standard negative binomial model when data are clustered.

Therefore, the negative binomial regression model was the best fit for the fracture data among the considered possible models based on AIC value and LR test. Table 5 presents the adjusted and unadjusted rate ratios (RaRs) from the negative binomial regression for fractures over 18 months. The advice arm was used as the reference group in the primary analyses for PreFIT. The unadjusted and adjusted fracture RaRs (95% Cis) for the exercise group compared to those randomised to advice were 1.11 (95% CI: 0.84, 1.46) and 1.20 (95% CI: 0.91, 1.59), respectively, indicating a statistically non-significant increase in fracture rate in the exercise group compared to advice only. For the comparison of MFFP with advice only, the unadjusted and adjusted fractures RaRs were 1.27 (95% CI: 0.97, 1.66) and 1.30 (95% CI: 0.99, 1.71), respectively, also indicating a statistically non-significant increase in fracture rate in the MFFP group compared to advice only.

**Table 5:** Adjusted and unadjusted rate ratios (RaRs) with 95% CI and *p*-values from the negative binomial regression for fractures over 18 months

Treatment comparisons^a^	Unadjusted		Adjusted^b^	
	**Rate ratio (95% CI)**	***p*-value**	**Rate ratio (95% CI)**	***p*-value**
Exercise vs Advice	1.11 (0.84, 1.46)	0.45	1.20 (0.91, 1.59)	0.08
MFFP vs Advice	1.27 (0.97, 1.66)	0.19	1.30 (0.99, 1.71)	0.06

^a^Advice is the reference group

^b^Adjusted for baseline covariates age, gender, deprivation score and log of baseline falls count. *CI* Confidence Interval

## Discussion

We explored the utility of several statistical models to fit fracture counts recorded within a large pragmatic clinical trial. Despite being the largest UK falls prevention trial to date, recruiting almost 10,000 older people and observing outcomes over 18 months, the PreFIT dataset had a low proportion of fall-related fractures overall and thus an excess of zero events. Fracture count data have distinct properties including positively skewed distribution, potential for recurrent fracture events, and multiple fracture events at one or more anatomical sites at time of injury.

We can summarise our findings as follows. Firstly, the zero-inflated and the hurdle models were not eligible to model fracture counts although our data had excess zeros. This is because zero fracture counts equate no fracture whereas zero-inflated models assume two zero generating processes and the hurdle models assumes ‘certain’ zeros. Secondly, based on the goodness-of-fit statistics, the standard Poisson model did not fit the data well. This was because there was data over-dispersion, but the Poisson model ignored this by assuming equality between mean and variance. The negative binomial model fitted the model better compared to the standard Poisson model as it accounted for the over-dispersion. The extended Poisson model and the extended negative binomial model, incorporating a random effect term to account for possible dependence among observations, did not give any gain over standard Poisson and standard negative binomial model, respectively. This suggests that the correlation between the two observations from the same cluster are negligible. Therefore, the negative binomial model fitted the fracture count data in the PreFIT dataset very well.

In our dataset, there were no missing fracture counts due to rigorous data collection using national hospital statistics and GP/radiographic records and triangulation approaches to confirm self-reported and routinely reported fracture events across the whole recruited sample. Therefore, we did not undertake sensitivity analysis. However, in practice, it is common to have missing counts. In such cases, one needs to consider sensitivity analysis under different missingness mechanisms to check the validity of model results. Our findings are based on this sample of community-dwelling older adults recruited from primary care thus may not apply to other clinical studies with much higher fracture event rates e.g. hospitalised or screened osteoporotic populations. We therefore encourage further work to externally validate our models.

In conclusion, after comparing four alternative statistical models for the analyses of fracture counts, we found that the negative binomial regression model was the best model to fit fracture counts with excess zeros within our large trial dataset of over 9000 older people. We recommend that this analytical approach should be considered for future population based studies recording fracture events.

## Data Availability

All reasonable requests for data should be sent to the corresponding author. Access to the available anonymised dataset may be granted following review by the research team.
